# Impacts of Interleukin-17 Neutralization on the Inflammatory Response in a Healing Ligament

**DOI:** 10.4172/2576-3881.1000113

**Published:** 2017-04-06

**Authors:** Anna EB Clements, Connie S Chamberlain, Ellen M Leiferman, William L Murphy, Ray Vanderby

**Affiliations:** 1 Department of Orthopedics and Rehabilitation, University of Wisconsin, Madison, Wisconsin, USA; 2 Department of Biomedical Engineering, University of Wisconsin, Madison, Wisconsin, USA

**Keywords:** Interleukin-17, Medial collateral ligament, Ligament healing, Immunomodulation

## Abstract

In this study, we sought to improve ligament healing by modulating the inflammatory response after acute injury through the neutralization of Interleukin-17 (IL-17), which we hypothesized would decrease inflammatory cell infiltration and cytokine production. Administration of an Interleukin-17 neutralizing antibody (IL-17 NA) immediately following a rat medial collateral ligament (MCL) transection resulted in alterations in inflammatory cell populations and cytokine expression within the healing ligament, but did not reduce inflammation. Specifically, treatment resulted in a decrease in M2 (anti-inflammatory) macrophages, an increase in T cells, and an increase in the levels of IL-2, IL-6, and IL-12 in the MCL 7 days post injury. IL-17NA treatment, and subsequent immunomodulation, did not result in improved ligament healing, as measured by collagen composition and wound size.

## Introduction

After injury, ligaments never regain their original mechanical or structural properties [1,2]. The repair process results in the formation of a neo-ligament, which is more scar-like in character than the native tissue. Early ligament healing is characterized by an infiltration of inflammatory cells including neutrophils and macrophages, followed by fibroblasts, myofibroblasts, and endothelial cells which together form granulation tissue and repair the wound through extracellular matrix (ECM) production *via* synthesis of type I and type III collagen, resulting in scar formation [3]. Macrophages infiltrate the injured ligament and produce matrix metalloproteinases (MMPs) as well as reactive oxygen and nitrogen species, which degrade collagen as part of the remodeling process and correlate to an increase in the size of the granulation tissue [3,4]. The degradation of healthy ligament and growth of the granulation tissue during remodeling corresponds to the localization of pro-inflammatory M1 macrophages [3]. Previous studies have shown non-specific depletion of macrophages does not improve ligament healing and is detrimental to early matrix formation and ligament strength [5]. In order to maintain the beneficial properties of the immune response while limiting the damaging effects of inflammation, we seek to modulate the immune response during ligament healing in order to improve ligament regeneration and reduce scar formation.

Interleukin-17 (IL-17) is a pro-inflammatory cytokine which promotes the recruitment of monocytes and neutrophils through an increase in local production of IL-8, monocyte chemoattractant protein-1 (MCP-1) and Groα, stimulates the production of hematopoietic cytokines including G-CSF and GM-CSF which expand the myeloid lineage, and stimulates IL-6 and PGE2 which enhance the local inflammatory environment [6]. IL-17 stimulates the production of IL-1β and TNF-α by macrophages [7], contributes to chronic inflammation associated with matrix destruction *via* inhibition of matrix production by chondrocytes and osteoblasts and activating the production of MMPs by macrophages, which ultimately leads to irreversible cartilage damage [8]. IL-17’s proven contribution to chronic inflammation and its inflammatory impact on cells of the myeloid lineage indicate that it may contribute to the acute inflammatory response after ligament injury.

TH17 cells are the main cell type responsible for IL-17 production, although neutrophils also express IL-17 and are prevalent in the early inflammatory phase of ligament healing [3,9]. While IL-17 expression is limited to a select group of cell types, the IL-17 receptor (IL-17 RA) is expressed on nearly every cell type, including fibroblasts and myeloid cells [8]. Previous microarray data indicate that there is an increase in IL-17 RA expression upon ligament injury, potentially enhancing the sensitivity of many cell types to IL-17 *via* IL-17 RA upregulation [10]. Recently, monoclonal antibodies against IL-17 or IL-17 RA have been used successfully in clinical trials for the treatment of diseases involving chronic inflammation. Their success indicates the potential for modulating other forms of IL-17 mediated inflammation, including the inflammatory phase of wound healing, with antibodies against IL-17 [8]. In this study, we sought to inhibit IL-17 activity after ligament injury through the application of a monoclonal antibody that neutralizes IL-17. We hypothesize that the application of an IL-17 neutralizing antibody (IL-17 NA) will modulate the immune response after ligament injury by reducing inflammatory cell populations and cytokine production within the ligament. Furthermore, modulating the inflammatory profile in an injured ligament will improve healing through the reduction of fibrosis and granulation tissue size. Alterations in the inflammatory response could improve ligament healing through modulation of ECM deposition in the granulation tissue and remodeling of the healthy ligament, specifically by increasing type I procollagen production, decreasing type III collagen deposition, and minimizing granulation tissue size.

## Materials and Methods

### Animal surgeries

This study was approved by the University of Wisconsin Institutional Animal Care and Use Committee. Six skeletally mature male Wistar rats (300–315 g) were used as an animal model for ligament injury and the medial collateral ligament (MCL) was chosen as a model for extra-capsular ligament healing.

Rats were separated randomly into 2 treatment groups (n=3 rats/ treatment group). All rats received a bilateral transection of the MCL while anesthetized *via* isofluorane. A 1 cm skin incision was made over the medial aspect of both the left and right stifles. The subcutaneous tissue was dissected to expose the gracilis muscle and underlying MCL. A full mid-substance transection of the MCL in the transverse plane was performed. Group 1 experimental animals received an interperitoneal (i.p.) injection in the lower right quadrant of 200 μL of 0.5 μg/μL murine IL-17 neutralizing antibody (R&D Systems, Minneapolis, MN) in sterile phosphate buffered saline (PBS) based on previous reports [11–15]. Another 20 μL was topically applied over each of the injured MCLs. Control animals underwent the same procedure with a murine IgG2A isotype control antibody (R&D Systems, Minneapolis, MN) at the same concentration in place of the IL-17 NA. Following transection and treatment, the muscular, subcutaneous, and subdermal tissue layers were each closed with 4-0 Dexon suture. All animals were allowed unrestricted cage movement immediately after surgery. Animals were sacrificed 7 days post-injury.

### Tissue collection and histology

Immediately upon sacrifice, the left MCL of each animal was dissected, measured, flash frozen into Optimal Cutting Temperature (OCT) compound, cut into 5 μm thick cryosections which were mounted on Superfrost Plus microscope slides and maintained at −70°C for future immunohistochemistry (IHC) and histology. The right MCL of each animal were dissected, measured, flash frozen, and stored at −70°C for later protein analysis.

**Figure 1:**
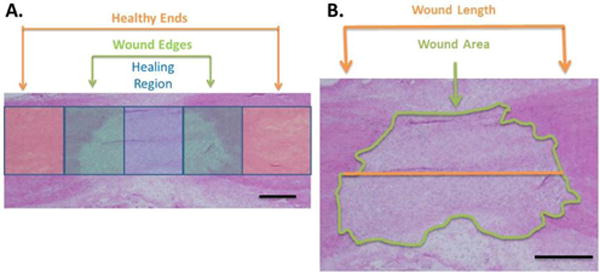
H&E staining of a healing MCL at day 7 post injury. A) All ligaments were divided into 3 regions for ECM and cell quantification; a healing region (granulation tissue), the wound edges, and the healthy ligament ends. Colored boxes represent the healthy ends (orange), wound edges (green) and healing region (blue); B) H&E stained section used for wound area and length measurements. The green line represents the encircled area of granulation tissue while the orange line indicates the length of the granulation tissue. Epiligament was not included in these measurements. Scale bars represent 500 μm.

Hematoxylin and eosin (H&E) staining was performed on tissue cryosections to observe the general morphology and granulation tissue size of the healing ligaments. Each ligament was divided into 3 regions based on the morphology; a healing region, wound edges, and the healthy ligament ends (Figure 1A) for cell counting and collagen quantification. H&E was also used to distinguish the border of the granulation tissue for wound width and area measurements (Figure 1B). Micrographs of each tissue section were taken using a camera assisted microscope (E6000 Nikon Eclipse microscope equipped with a DP79 Olympus camera) and the granulation tissue dimensions were measured using Image J for 3 tissue sections per animal. Length measurements were taken at the longitudinal midpoint of the ligament. Cross sectional area measurements of the granulation tissue did not include the epiligament tissue.

### Immunohistochemistry (IHC)

Immunohistochemistry (IHC) was performed on cryosections of the MCL specimens to identify ECM components and specific cell types. ECM components were stained using mouse monoclonal antibodies to Type 1 Procollagen (undiluted; SP1.D8; Developmental Hybridoma, Iowa City, Iowa) and Type III Collagen (diluted 1:8000; Sigma-Aldrich, St Louis, Missouri). Cell types were characterized using mouse monoclonal antibodies to CD68 (M1 macrophages), CD163 (M2 macrophages), α-Smooth Muscle Actin (myofibroblasts) and CD3 (T-cells). These antibodies were obtained from Abcam-Serotec (Raleigh, NC) and were used at a dilution of 1:100. For IHC, cryosections were thawed, fixed in acetone, incubated in 3% hydrogen peroxide to eliminate endogenous peroxidase activity, blocked with background buster (Innovex Biosciences, Richmond, CA) for 30 minutes, and then incubated with the antibody of interest for 2 hours. Following primary antibody incubation and rinsing off unbound antibody, sections were incubated with biotin, followed by incubation in streptavidin-conjugated horseradish peroxidase (Stat Q staining kit from Innovex Biosciences, Richmond, CA). Bound antibodies were visualized with diaminobenzidine (DAB), dehydrated, cleared, and coverslipped for light microscopy.

Micrographs were taken at 400 X in each area of interest (both proximal and distal for wound edges and healthy ligament ends) for 3 sections of each animal for M1 macrophages (ED1+), M2 macrophages (ED2+), myofibroblasts, type 1 procollagen, and type III collagen. Positively stained cells were counted in each micrograph for M1, M2, and myofibroblasts. Cell counts for 3 sections of the same animal were summed together and expressed as cells per ligament area. Image J was used to threshold DAB staining of the ECM proteins and was used to quantify the percent of the total area that was positively stained in each region for 3 sections of each animal, which were then averaged together. T cell counts were made for the 3 entire cryosections of each MCL to compensated for the low number of positively stained cells in each ligament. T cells were also counted in the entire granulation tissue area for the same purpose. Student T-Tests (two tailed, equal variance) were performed using Microsoft Excel to assess statistical differences between treatment groups. A p-value ≤ 0.05 was considered significant.

### Cytokine measurements

A rat 10-plex Luminex assay (Life Technologies, Grand Island, NY) for IL-1α, IL-1β, IL-10, IL-2, IL-12, IL-4, IL-6, tumor necrosis factor-α (TNF-α), inferon-γ (IFN-γ), and granulocyte macrophage-colony stimulating factor (GM-CSF) was run to measure the cytokine levels in the MCLs of each treatment group. Dissected MCLs were washed in Cell Wash Buffer (Bio-Rad, Hercules, CA), and were homogenized in Navy Bead Lysis Kit tubes containing 0.9–2.0 mm stainless steel bead blend, 3.2 mm steel balls (Next Advance, Averill Park, NY) and Lysing Solution (Bio-Rad, Hercules, CA) using a Bullet Blender (Next Advance, Averill Park, NY) for 10 minutes. The supernatant was collected, pooled for the same treatment condition (n=3 MCLs), and use for the 10-plex assay. The 10-plex assay was prepared and performed according to the manufactures instructions. Standards, MCL samples (n=3), rat spleen (positive control), and cell lysis buffer (negative control) were measured on a Luminex 200 instrument (Luminex, Austin, TX). Cytokine concentrations were measured and converted to total cytokine mass by multiplying by the volume of the supernatant collected after cell lysis. In order to be considered above the detection limit, only cytokine levels within the standard curve and at least 2 standard deviations above the background were considered. These criteria eliminated IL-1α, IL-4, TNF-α, IFN-γ, GM-CSF, from analysis because their concentration was not detectable using this assay. Student t-Tests (two-tailed, equal variance) assessed statistical differences between treatment groups. A p-value ≤ 0.05 was considered significant.

## Results

### Immune cell profile

**Figure 2:**
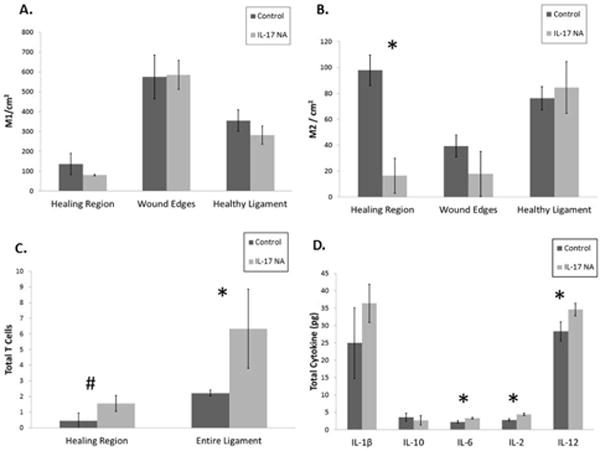
Inflammatory cell counts and cytokine expression. A) IL-17NA treatment did not significantly affect M1 pro-inflammatory macrophage numbers compared to control in any region of the ligament; B) IL-17NA treatment significantly (^*^p=0.001) decreased M2 anti-inflammatory macrophage numbers in the healing region compared to IgG controls; C) IL-17 NA treatment significantly (^*^p=0.05) increased the total number of T cells in the MCL and there was a trend (^#^p=0.06) of an increase in T cell numbers in the healing region also compared to IgG controls. Values for cell counts are expressed as mean cell count per area ± S.D; D) Total mass of cytokines in 3 pooled MCLs for each treatment group. Treatment with IL-17NA significantly increased the total mass of IL-2 (^*^p=0.004), IL-6 (^*^p=0.014), and IL-12 (^*^p=0.03) in 3 pooled MCLs of each treatment group. Levels of IL-1β and IL-10 were unaffected. Values for cytokine expression represent mean of total cytokine mass±S.D.

To determine the impact of IL-17 neutralizing antibody on the immune cell population in an injured MCL, we analyzed the number of M1 macrophages, M2 macrophages, and T cells in the different regions of the MCL with IHC. We conducted cell counts of M1 and M2 macrophages for each region of the MCL. There was no significant change in the number of M1 macrophages in any region of the healing ligament upon Il-17 NA administration (Figure 2A). Treatment with IL-17 NA at the time of injury significantly (p=0.001) decreased the number of M2 macrophages in the healing region of the MCL (Figure 2B). In contrast, treatment with IL-17 NA significantly (p=0.05) increased the number of T cells in the entire MCL (Figure 2C).

### Cytokine levels

We measured the levels of 10 different pro and anti-inflammatory cytokines in the injured MCLs of IL-17 NA or IgG treated animals 7 days after injury using a cytokine multiplex assay. Levels of IL-1β, IL-2, IL-6, IL-10, and IL-12 were detectable in the pooled MCLs using the multiplex assay. Treatment with IL-17 NA significantly increased IL-2, IL-6, and IL-12 levels (p=0.004, p=0.014, and p=0.03 respectively), all of which are associated with T cell activity, while the amount of anti-inflammatory IL-10 (p=0.44) and pro-inflammatory IL-1β (p=0.16) were not affected by IL-17 NA treatment (Figure 2D).

### Wound size and collagen composition

**Figure 3:**
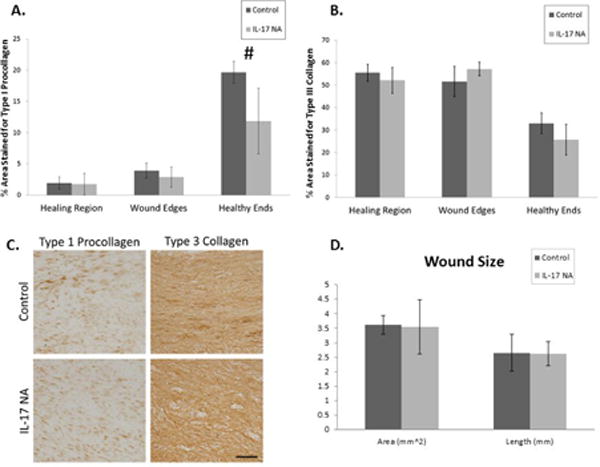
Granulation tissue characteristics. A) No significant change in the production of Type 1 Procollagen was observed in any region, however there was a trend (^#^p=0.07) of less Type 1 Procollagen in the healthy ends of the IL-17 NA treated MCLs; B) Type III Collagen was not significantly different between treatment groups at any location. Values for collagen expression represent the percent of stained tissue area ± S.D; C) IHC of Type 1 Procollagen and Type III Collagen in the healing region of IL-17NA treated and IgG control MCLs, scale bars represent 50 μm. No significant difference was observed in fibrosis, as indicated by high levels of Type III Collagen in the granulation tissue; D) Wound size measurements for both IL-17NA treated and Control (IgG) MCLs. There was no significant difference for either wound area or length between treatment groups. Wound size values represent the mean wound area or length±S.D.

We distinguish the granulation tissue from the healthy ligament tissue with H&E staining. Measurements of the 2 dimensional wound area and the wound length (mid-ligament, parallel to the long axis) indicate that treatment with an IL-17 neutralizing antibody does not impact wound size 7 days post injury (Figure 3D). IHC staining for type I procollagen and type III collagen examined extracellular matrix composition. Type I procollagen, the precursor to type 1 collagen is a hallmark of more regenerative healing in ligament and is the major ECM component in normal MCL, while type III collagen is associated with scar formation in ligament after injury.[3] IL-17 NA did not significantly alter either type 1 procollagen or type III collagen in any region of the ligament (Figures 3A–3C). However, IL-17 NA tended to (p=0.07) decrease type I procollagen in the healthy ligament ends (Figure 3B).

## Discussion

The purpose of this study was to evaluate the impact of neutralizing IL-17 in a healing ligament. We hypothesized that blocking IL-17 activity with a neutralizing antibody would impact the inflammatory response and reduce inflammatory cell populations and cytokine production within the healing ligament. Our results indicated that administration of IL-17 NA at the time of ligament injury significantly decreased the number of M2 macrophages in the granulation tissue, significantly increased the number of T cells in the ligament, and significantly increased the levels of IL-2, IL-6, and IL-12 in the MCL 7 days post injury. These results collectively indicate that we affected IL-17 activity during the inflammatory phase of ligament healing through the administration of a neutralizing antibody and that IL-17 inhibition impacts inflammatory cell populations. However, the effect was not exclusively anti-inflammatory. IL-17 NA treatment significantly decreased the number of M2 alternatively activated, anti-inflammatory macrophages in the healing region of the ligament. Although M2 macrophage populations were decreased in the granulation tissue, the amount of IL-10 (an anti-inflammatory cytokine that is known to be produced by M2 macrophages and to induce the M2 phenotype did not change [16]. The disparity between M2 macrophage numbers and IL-10 protein levels may be due to the IL-10 produced by M2 macrophages in other regions of the ligament, such as the healthy ends or epiligament, which could wash out any changes in IL-10 levels in the granulation tissue alone.

IL-17 neutralization selectively modulated the M2 macrophage phenotype rather than the M1 macrophage phenotype. The reduction in M2 macrophages by IL-17NA during ligament healing is a new finding to our knowledge. Previous studies indicate that the M2 population increased *via* proliferation rather than recruitment from the blood [17]. The decrease in M2 macrophages may be due to the ability of IL-17NA to inhibit macrophage proliferation rather than recruitment in a healing ligament. The reduced number of M2 macrophages may also contribute to the increase in T-lymphocytes. The arginase pathway predominates L-arginine metabolism in M2 macrophages, which results in T-lymphocyte suppression [18,19]. A decrease in M2 macrophage numbers would therefore reduce the suppression of T-lymphocyte proliferation. Increased IL-12, IL-6, and IL-2 production after IL-17 NA treatment supports the changes observed in T lymphocyte numbers, as each of these cytokines contributes to T lymphocyte proliferation or expansion [20–22]. However, macrophages are the major source for IL-6 and IL-12, which makes the significant increase of these cytokines surprising after we observed no change in M1 macrophage numbers and a decrease in M2 macrophage numbers. Previous research demonstrates that IL-17 modulated monocyte/macrophage recruitment whereas neutralization of IL-17 significantly reduces monocyte migration during autoimmune-derived inflammatory diseases, such as rheumatoid arthritis [23]. Additionally, research has shown that IL-17 contributed inflammation intensified with increased T-cell involvement [24]. Unlike an autoimmune associated disease, MCL injury typically contains few T cells within the tissue, which partially explains why IL-17 neutralization did not inhibit inflammation in this model. Instead, IL-17 may play a greater role in adaptive and/or autoimmune-modulated reactions, which are associated with a high level of T cell involvement, compared to innate immune mediated responses, which exhibit low T cell presence. It is also possible that the concentration of the IL-17 NA was not sufficient to inhibit the majority of IL-17 activity at the time of inflammatory macrophage recruitment, 2–5 days post injury (3).

Treatment with IL-17NA significantly impacts both the inflammatory cell population and cytokine levels within a healing MCL. Despite this modulation, measurements of wound size and composition indicate that regenerative healing was neither induced nor impaired. While IL-17 has been shown to be a therapeutic target for treatment of chronic inflammation, it may not play a significant role in the acute inflammation of ligament healing. However, it may be of more interest to study further the role of IL-17 in M2 macrophage and T cell proliferation in future studies.

This report is not without limitations. First, the experiment only examined one IL-17 NA concentration, time of dosing, day of healing, when optimally, additional experimental perturbations would be included. Experimental perturbations were based on our previous research and/or previous reports [3,11–15,25–27]. Second, the number of animals used for each treatment group was low. However, we were able to report significance from this number of animals. Future studies will include greater experimental examination of IL-17 NA on both biological and functional healing.
